# Transverse occiput position: Using manual Rotation to aid Normal birth and improve delivery OUTcomes (TURN-OUT): A study protocol for a randomised controlled trial

**DOI:** 10.1186/s13063-015-0854-3

**Published:** 2015-08-18

**Authors:** Bradley de Vries, Hala Phipps, Sabrina Kuah, John Pardey, Joanne Ludlow, Andrew Bisits, Felicity Park, David Kowalski, Jon A. Hyett

**Affiliations:** RPA Women & Babies, Royal Prince Alfred Hospital, Missenden Rd, Camperdown, Sydney, NSW 2050 Australia; Discipline of Obstetrics, Gynaecology and Neonatology, University of Sydney, Sydney, NSW 2006 Australia; Women’s and Children’s Hospital, Department of Perinatal Medicine, 72 King William Road, North Adelaide, SA 5006 Australia; Nepean Hospital, Department of Obstetrics and Gynaecology, Derby St., Penrith, NSW 2750 Australia; Royal Hospital for Women, Maternity Division, Barker Rd, Randwick, Sydney, NSW 2031 Australia; The John Hunter Hospital, Department of Obstetrics and Gynaecology, Lookout Rd, New Lambton, Newcastle, NSW 2035 Australia; Canterbury Hospital, 575 Canterbury Rd, Campsie, Sydney, NSW 2194 Australia

**Keywords:** Transverse position, Caesarean section, Fetal malposition, Anterior position, Operative delivery, Instrumental delivery, Abdominal ultrasound, Manual rotation, Digital rotation, Second stage of labour

## Abstract

**Background:**

Fetal occiput transverse position in the form of deep transverse arrest has long been associated with caesarean section and instrumental vaginal delivery. Occiput transverse position incidentally found in the second stage of labour is also associated with operative delivery in high risk cohorts. There is evidence from cohort studies that prophylactic manual rotation reduces the caesarean section rate. This is a protocol for a double blind, multicentre, randomised, controlled clinical trial to define whether this intervention decreases the operative delivery (caesarean section, forceps or vacuum delivery) rate.

**Methods/Design:**

Eligible participants will be ≥37 weeks pregnant, with a singleton pregnancy, and a cephalic presentation in the occiput transverse position on transabdominal ultrasound early in the second stage of labour. Based on a background risk of operative delivery of 49%, for a reduction to 35%, an alpha value of 0.05 and a beta value of 0.2, 416 participants will need to be enrolled. Participants will be randomised to either prophylactic manual rotation or a sham procedure. The primary outcome will be operative delivery. Secondary outcomes will be caesarean section, significant maternal mortality and morbidity, and significant perinatal mortality and morbidity.

Analysis will be on an intention-to-treat basis. Primary and secondary outcomes will be compared using a chi-squared test. A logistic regression for the primary outcome will be undertaken to account for potential confounders. This study has been approved by the Ethics Review Committee (RPAH Zone) of the Sydney Local Health District, Sydney, Australia, (protocol number: X110410).

**Discussion:**

This trial addresses an important clinical question concerning a commonly used procedure which has the potential to reduce operative delivery and its associated complications. Some issues discussed in the protocol include methods of assessing risk of bias due to inadequate masking of a procedural interventions, variations in intervention efficacy due to operator experience and the recruitment difficulties associated with intrapartum studies.

**Trial registration:**

This trial was registered with the Australian New Zealand Clinical Trials Registry (identifier: ACTRN12613000005752) on 4 January 2013.

## Background

Fetal occiput transverse (OT) position in the form of deep transverse arrest has long been associated with caesarean section and instrumental vaginal delivery [1]. OT position incidentally found in the second stage of labour is also associated with operative delivery in high risk cohorts [2, 3]. Caesarean section is now a major contributing factor to maternal mortality and morbidity following childbirth in developed countries [4, 5]. Obstetric intervention by forceps and vacuum delivery is associated with complications to the maternal genital tract and neonate, respectively [6–8].

Manual rotation from the OP to the occiput anterior (OA) position is a safe, relatively simple and easy to perform procedure which could reduce the operative delivery rate (defined as vacuum delivery, forceps delivery and/or caesarean section), and therefore increase the chances of a normal vaginal birth [9]. It is performed by only a minority of obstetricians and midwives in Australia and New Zealand, and yet is considered to be acceptable by the vast majority [10, 11]. However, obstetricians and midwives would perform a manual rotation if there was evidence that it resulted in an absolute risk reduction of about 18% [10, 11], suggesting that demonstration of efficacy will translate into clinical practice. One cohort study with a mixed population of OT and occiput posterior (OP) positions showed promising results [12], but there has been no randomized controlled trial of attempted manual rotation for OT position in the second stage of labour.

### Epidemiology

The prevalence of OT position is 19 to 49% at the onset of labour [13, 14], 10 to 20% in the second stage of labour [3, 14–16] and 3 to 8% at delivery [13, 17]. In one prospective cohort, the operative delivery rate was 87% when OT position was present at birth, compared with 24% when the fetus was in the more common OA position [13]. When the OT position was present at the beginning of the second stage of labour, the operative delivery rate was 49% compared with 31% when the position was OA [3]. The association between OT position in the second stage labour and operative delivery is maintained after adjusting for multiple confounders, including ethnicity, parity, epidural use, abnormal second stage Cardiotocograph and maternal age (odds ratio for OT versus OA: 2.0, 95% confidence interval: 1.1 to 3.7) [3].

Thus, of all women who plan a normal birth, 10 to 20% will have a fetus in the OT position early in the second stage of labour. These women will be eligible to have a manual rotation to modify their background risk (of approximately 50%) of intervention with forceps, vacuum or caesarean section.

### Complications of occiput transverse position

The OT position is associated with more frequent augmentation of labour, episiotomy, third or fourth degree perineal trauma, febrile morbidity, prolonged second stage of labour and low five-minute Apgar test score [2].

### The intervention in current practice

Manual rotation is a well-accepted component of obstetric practice, particularly in the context of rotating the fetus to the OA position immediately prior to the application of non-rotational forceps, such as Neville-Barnes [18]. However, it is also used in a prophylactic setting (without assisted delivery) to reduce the complications associated with OT delivery [12]. In a survey of obstetricians in Australia and New Zealand, 70% believed it was acceptable in a prophylactic setting; however, only 38% had performed a manual rotation in the last year, and most of these had only performed one or two [10]. Both obstetricians and midwives reported they would perform a manual rotation if there was an 18% absolute risk reduction in operative delivery [10, 11]. Thus demonstration of efficacy would provide substantial scope for the intervention to be introduced into widespread practice.

### The efficacy of the intervention

There is only one study which assessed the efficacy of manual rotation for OT position in the second stage of labour [12]. This retrospective database cohort study reported on both OT and OP positions as a single group, and found a 9% risk of caesarean section when manual rotation was performed compared with a 41% risk when it was not [12]. The authors had information on the fetal position at the time of birth, but not earlier in the second stage of labour when the procedure was performed. Thus OP fetuses that were destined to rotate naturally to the OA position would have been included in the intervention group, but not the control group, which would result in an overestimation of the caesarean section rate in the control group and of the efficacy of manual rotation. The authors did not report on the success of manual rotation for OT and OP positions separately.

### The safety of the intervention

Manual rotation has long been considered to be safe [9]. One retrospective cohort study reported lower rates of complications when it was performed for OP position compared to when it was not (Table 1) [12].

**Table 1:** Complications of manual rotation versus expectant management (data from Shaffer *et al*.) [12]

Complication	Manual rotation (n = 731)	Expectant (n = 2,527)	Adjusted OR (95% CI)
Post-partumhaemorrhage	22.3%	33.1%	0.78 (0.62-0.98)
3rd and 4th degree tears	15.7%	20.1%	0.64 (0.47-0.88)
Cervical laceration	2.2%	1.0%	2.46 (1.11-5.44)
Chorioamnionitis	8.6%	14.4%	0.68 (0.50-0.92)
Endometritis	3.6%	7.2%	1.25 (0.75-2.10)
5-minute Apgar score <7	1.8%	3.7%	0.50 (0.26-0.94)
Umbilical cord arterial pH <7	0.6%	1.4%	0.55 (0.15-2.01)
Base excess < −12	3.5%	3.2%	1.21 (0.64-2.30)
Shoulder dystocia	2.1%	1.1%	1.61 (0.73-3.56)
Birth trauma	1.09%	1.23%	0.50 (0.20-1.26)

Thus incidence of third and fourth degree tears, chorioamnionitis, post-partum haemorrhage, endometritis and 5-minute Apgar score of less than seven all improved when prophylactic manual rotation was performed but cervical laceration was increased. In the TURN-OUT trial, manual rotation will be performed at full dilatation that theoretically will minimize the risk of cervical laceration.

There is also a single case report of an umbilical cord prolapse associated with a manual rotation [19]. In this report, an emergency caesarean section was performed and the baby was born alive and presumably well. Other risk factors such as amniotomy, application of a fetal scalp electrode and external cephalic version were more frequently associated with umbilical cord prolapse [19].

### The timing of the intervention

Manual rotation from the OP position may be performed at full cervical dilatation or late in the first stage of labour. In a French case control study (n = 147) in a labour ward where prophylactic manual rotation was performed routinely, two risk factors for inability to rotate the fetus were identified: (1) attempted rotation before full dilatation and (2) failure to progress in labour [20]. Thus we consider that it would be reasonable to attempt prophylactic manual rotation after full dilatation is achieved, but relatively early in the second stage of labour, before the fetal head becomes impacted in the maternal pelvis.

### Rationale for operative delivery as the primary outcome

Operative delivery was selected as the primary outcome for the TURN-OUT trial because it is clearly associated with important short and long term outcomes for the woman and her baby [6–8, 21–24]. Other important obstetric parameters will be measured, but will be reported as secondary outcomes. Reducing the rate of operative delivery for OP position is perceived to be very important by obstetricians and midwives [10, 11]. In high income countries, emergency caesarean section is associated with significant maternal morbidity and a five-fold increase in maternal mortality [25].

### Explanation for choice of comparator

A sham procedure was chosen as a comparator to minimize the risk of performance bias. There would be substantial scope for management to differ according to treatment allocation if it was known. For example, a women could be encouraged to push more strongly if her midwife was aware that a manual rotation had been performed.

## Methods/Design

### Aim and hypothesis

The aim of this study is to determine the efficacy of elective manual rotation in the management of OT position in the second stage of labour. We hypothesise that, among women who are at least 37 weeks gestation whose baby is in the OT position early in the second stage of labour, manual rotation compared with a sham rotation will result in a reduction in operative delivery.

The primary objectives are to determine differences between intervention and control groups in operative delivery rate (defined as vacuum, forceps and/or caesarean section deliveries). The secondary objectives are to determine differences between intervention and control groups in: caesarean section, combined measure of serious maternal morbidity and mortality within six weeks of birth, and combined measure of serious perinatal and neonatal morbidity and mortality within six weeks of birth.

### Trial design

The TURN-OUT trial is designed as a superiority, double blind, multicentre, randomised, controlled clinical trial with two parallel groups and a primary endpoint of fetal mode of delivery at birth. Randomization will be performed as block randomization with a 1:1 allocation.

## Methods/Design

### Study settings

The study will recruitment hospitals in Australia which have 2,000 or more deliveries per year, namely: Canterbury Hospital (NSW), The John Hunter Hospital (NSW), The Nepean Hospital (NSW), The Royal Hospital for Women, Randwick (NSW), The Royal Prince Alfred Hospital (NSW) and The Women and Children’s Hospital (SA). We do not intend to recruit in any other centres. The intervention will be performed by obstetricians or midwives who are experienced in performing a manual rotation.

### Eligibility criteria

#### Inclusion criteria

The inclusion criteria for the study are as follows:

1. aged ≥18 years,
2. singleton pregnancy,
3. ≥37 weeks of gestation,
4. planned vaginal birth,
5. cephalic presentation,
6. full cervical dilatation,
7. occiput transverse position confirmed by ultrasound where the occiput is <45° from the transverse plane.

#### Exclusion criteria

Most exclusion criteria were selected on the basis of predisposition to requiring an operative delivery, and are as follows:

1. clinical suspicion of cephalopelvic disproportion,
2. previous caesarean section,
3. brow or face presentation,
4. ‘Pathologic’ CTG according to Royal College of Obstetricians and Gynaecologists classification plus either baseline >160 beats per minute or reduced variability,
5. fetal scalp pH <7.25 or lactate >4 mmol/L,
6. known or suspected chorioamnionitis,
7. intrapartum haemorrhage >50 mL,
8. temperature ≥38.0°C in labour,
9. pre-existing maternal diabetes,
10. suspected fetal bleeding disorder (theoretical risks associated with procedures involving manipulation of fetal position),
11. known major anatomical fetal abnormality (could influence safety or efficacy of manual rotation).

Study centres must be able to provide a 24 hour on-call service, with experienced operators to perform the intervention, in order to be included in the study.

### Intervention

Manual rotation is performed at full dilatation (when the woman has the first urge to push or after one hour, whichever occurs first) if the fetal position is OT. The technique employed will be at the discretion of the operator performing the procedure. The intervention will be performed by obstetricians or midwives who are experienced in performing a manual rotation and have performed at least 20 procedures. All operators will complete a questionnaire outlining their technique and experience.

With the membranes ruptured, a vaginal examination is performed and the woman is asked to bear down. Constant pressure is exerted with the index finger against the lambdoid suture to rotate fetal head. This may take two to three contractions and the position is commonly held for two contractions while the woman bears down to reduce the risk of reverting back to the OP position. Alternatively, the examiner places two fingers behind the fetal ear or the entire hand behind the occiput and applies constant flexion and rotation to the fetal head.

For the purposes of the TURN-OUT Trial, the procedure will be described as a ‘digital rotation’ if only the fingers are used, and as a ‘manual rotation’ if the whole hand is used.

### Comparator

Once full dilatation has been diagnosed (when the woman has the first urge to push or after one hour, whichever occurs first), women randomized to the sham rotation will have the same apparent vaginal examination as the intervention, but no rotational force will be applied. The woman is asked to bear down. The accoucheur places fingers in the vagina over five contractions as if they were performing a manual rotation.

### Criteria for discontinuing or modifying the intervention

The intervention or sham will be discontinued if there is a clinical necessity or at the request of the participant. This could occur if there is evidence of fetal compromise necessitating emergent delivery or if the participant is in significant discomfort. Each operator will complete a data collection form at the time of the procedure or sham, which will describe in detail what was done. Adherence with treatment allocation will be monitored by comparing these datasheets with the computer randomisation records. All interventions and usual care provided by doctors and midwives looking after the participant will be allowed. However, if the doctor is intending to perform an operative delivery or a manual rotation, the woman will not be randomised. Data will be collected about use and timing of any manual rotations performed by the participant’s clinician.

### Outcomes

#### Primary outcome

The primary outcome will be operative delivery (vacuum, forceps and/or caesarean section). Operative delivery will be performed at the discretion of the clinicians caring for the woman who will be blinded to the treatment allocation. In Australia, most obstetricians perform caesarean section and instrumental deliveries in line with the ACOG recommendations [10]. That is, operative delivery for prolonged second stage of labour is considered after one hour of full dilatation for parous women without an epidural, two hours for parous women with an epidural or nulliparous women without an epidural, and three hours for nulliparous women with an epidural. Operative delivery is also performed for suspected fetal compromise manifested as a pathological cardiotocograph (especially prolonged decelerations, variability <5 for more than 90 minutes or baseline >160 beats per minutes), fetal scalp lactate >4.8 mmol/L or pH <7.20.

#### Secondary outcomes

The secondary outcomes will be as follows:

1. Caesarean section (reported as proportion of participants who had a caesarean section).
2. Serious maternal morbidity or mortality (combined outcome). This will include one or more of the following: post-partum haemorrhage requiring blood transfusion; third or fourth degree perineal tears; dilatation and curettage for bleeding or retained placental tissue; cervical laceration; vertical uterine incision; vulvar or perineal haematoma; pneumonia; venous thromboembolism requiring anticoagulation; wound infection requiring hospital stay more than seven days; readmission to hospital for obstetric-related causes; wound dehiscence; maternal fever of at least 38.5°C on two occasions at least 24 hours apart (not including the first 24 hours); bladder, ureter or bowel injury requiring repair; genital tract fistula; bowel obstruction; and/or admission to intensive care unit. This will be reported as a proportion of participants with serious morbidity or mortality.
3. Serious perinatal and neonatal morbidity and mortality within six weeks of birth (combined outcome). This will include one or more of the following: shoulder dystocia requiring manoeuvres other than McRoberts manoeuvre or suprapubic pressure, or resulting in neonatal injury; five-minute Apgar score <4; arterial cord pH <7.0 or lactate >10 mmol/L or base excess <−15; serious birth trauma, “seizures within 24 hours of birth, intubation and ventilation for >24 hours, tube feeding for more than four days; admission to neonatal intensive care for more than four days; and/or neonatal jaundice requiring phototherapy. This will be reported as the proportion of participants with serious morbidity and/or mortality.
4. Prolonged second stage of labour, defined as:
  - more than one hour for parous women without epidural analgesia,
  - more than two hours for nulliparous women without epidural analgesia or parous women with epidural analgesia, or
  - more than three hours for nulliparous women with epidural analgesia.

#### Other outcomes

Other outcomes will be assessed during delivery admission and at six-weeks, six-months and one-year postpartum. The following outcomes will be assessed during delivery admission:

1. length of second stage (median),
2. Time from randomization until delivery (median),
3. time from intervention or sham until delivery (median),
4. estimated blood loss at delivery (median: visual estimation by midwife or doctor),
5. any perineal or vaginal trauma requiring suturing (proportion),
6. length of hospital stay (median),
7. for operative delivery, outcomes will be reported according to indication (prolonged second stage, suspected fetal compromise and other):
  - prolonged second stage defined as above;
  - suspected fetal compromise, defined as the presence of a pathological CTG according to National Institute of Health and Clinical Excellence guidelines, a fetal scalp lactate >4.8 mmol/L or a fetal scalp pH <7.2;
  - operative delivery in the presence of both prolonged second stage of labour and suspected fetal compromise will be classified as operative delivery for prolonged second stage;
  - operative delivery in the absence of both prolonged second stage of labour and suspected fetal compromise (such as maternal request or maternal exhaustion) will be classified as other.

The following outcomes will be assessed at six weeks:

1. still breast feeding (proportion),
2. satisfaction with birth (visual analogue scale) (median),
3. saw a health professional for depression since delivery (proportion), and
4. health-related quality of life (Short Form-12 (SF-12)) (median).

The following outcomes will be assessed at six months:

1. still breast feeding (proportion),
2. saw a health professional for depression since delivery (proportion), and
3. Health-related quality of life (SF-12) (median).

The following outcomes will be assessed at one year:

1. still breast feeding (proportion),
2. saw a health professional for depression since delivery (proportion),
3. Health-related quality of life (SF-12) (median), and
4. pelvic floor function (bowel, urinary, prolapse and sexual function domains) measured using the Australian pelvic floor function questionnaire [26] (medians).

### Sample size

The sample size (416) was calculated on the basis of the primary outcome. This power calculation is based on our prospective cohort of women with a fetus in the OT position [3], and our surveys of obstetricians and midwives who would perform a manual rotation if it reduced the rate of operative delivery by about 18% [10, 11]. To detect a reduction in operative delivery from 49% in controls to 35% in the intervention group, a sample size of 208 in each group (total = 416) will be required for α = 0.05 (two-tailed), β = 0.20 (power = 80%) (calculated using Epi Info™ version 3.3.2, Centers for Disease Control and Prevention, Atlanta, USA).

### Randomization and allocation concealment

Randomization will be stratified by parity, hospital site and epidural due to the potentially strong association between operative delivery (the primary outcome) and each of these factors. Randomization will be centrally controlled using computerized sequence generation, which can be accessed 24 hours per day using a toll-free telephone line.

In order to reduce the risk of randomising an ineligible participant, randomisation will occur immediately before the intervention or sham procedure is to be performed. An example of a participant becoming ineligible would be if the fetus rotated from the OT to the OA position. Each investigator will complete a data collection form at the time the manual rotation or sham procedure is performed, outlining the treatment allocation, clinical findings and whether or not the fetus was successfully rotated.

### Blinding

The following groups will be masked: the participants, the clinicians caring for the participant (including doctors and midwives), the data collectors and the statisticians who will perform the analysis. Unblinding will occur if the clinician requests it on the basis of clinical need or if the participant insists.

### Data collection, management and analysis

#### Study conduct

**Fig. 1:**
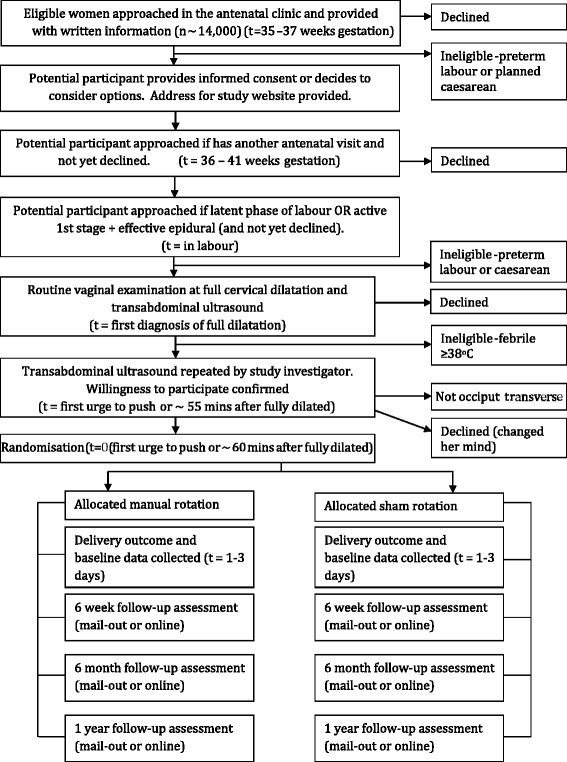
An overview of the conduct of the TURN-OUT trial. Losses to follow-up for the primary outcome are not expected, as it occurs within hours of randomisation and the mode of delivery (caesarean, vacuum, forceps and normal birth) is easily obtained from the hospital records

Consent will occur at three possible time points (Fig. 1): antenatal, in the latent phase of labour or in the active phase of the first stage of labour, with an effective epidural anaesthesia. Participants will be provided with written information via information pamphlets, posters and the trial website. Informed consent will be obtained by research midwives or midwives and/or medical staff involved in potential participants’ care (Fig. 1, Table 2). A detailed information sheet will be provided to all participants. Participants will be informed of the potential risks of manual rotation, including umbilical cord prolapse, given the opportunity to ask questions and informed that they have the right to change their mind at any time.

**Table 2:** The TURN-OUT study timeline for the schedule of enrolment, allocation and follow-up

	Enrolment		Allocation	Post-allocation				
Time point	35-37 weeks gestation	1st stage labour	During 2nd stage of labour	Immediately after allocation	1-3 days	6 weeks	6 months	12 months
1st eligibility screen	X							
2^nd^ eligibility screen		X						
Informed consent	X	X						
Allocation			X					
Intervention				X				
Assessments:								
Labour and delivery			X	X	X			
Operative delivery					X			
Perineal outcome					X			
Blood loss					X			
Maternal complications					X	X		
Hospital stay					X			
Readmission						X		
Neonatal outcomes					X	X		
Neonatal ICU admission(s)					X	X		
Satisfaction with birth						X		
Breastfeeding						X	X	X
Health-related quality of life						X	X	X
Pelvic floor						X	X	X
Depression						X	X	X

An ultrasound will be performed at full dilatation by the clinician caring for the woman, and the findings will be recorded on a data sheet immediately afterwards. An hour after full dilatation or at the first urge to push, a study investigator (with no clinical responsibility for the woman in the trial) will confirm the OT position by a second (pre-procedure) bedside ultrasound. If the fetal position is still OT and the woman still wishes to participate then the study investigator will randomise the woman to either manual or sham rotation. The treatment allocation will be recorded on a randomisation sheet, which the investigator will keep on their person and not show to any of the participant’s medical staff. After the manual rotation or sham has been performed the ultrasound will be repeated, ensuring that the woman and her medical staff do not see the screen. The investigator will leave and the woman will have her usual care from this point onwards. The investigator will record the findings on vaginal examination, details of the procedure and post-procedure ultrasound findings on the same data sheet as the pre-procedure ultrasound. The study investigator will keep this data sheet on their person and not show it to any of the participants’ medical staff.

#### Outcomes

Mode of delivery will be ascertained from the medical records to measure the primary outcome. Labour and delivery outcomes, perineal trauma, blood loss, duration of hospitalisation, short term neonatal outcomes, admission to the neonatal ICU, maternal or neonatal readmission to the same institution and other components of the combined secondary outcomes will be ascertained by a study investigator not involved in clinical care, using the medical records and by contacting participants’ clinicians for further information if required. Maternal depression, health-related quality of life (SF-12), birth satisfaction (visual analogue score), maternal or neonatal readmission to another institution, ongoing breastfeeding, pelvic floor symptoms and components of the combined secondary outcomes will be collected by structured maternal questionnaires at six weeks, six months and 12 months post-delivery, as outlined in section Outcomes. Questionnaires will be completed by mail-out, online via the trial website and by telephone, depending on the participants’ preferences (Fig. 1, Table 2). Data collectors will be unaware of the treatment allocation at all times.

As the primary outcome is mode of delivery and randomisation occurs during the second stage of labour, we expect 100% ascertainment for the primary outcome. Study investigators will perform site visits about four times per year to promote recruitment, provide education for clinical staff and site investigators and audit of centre medical records to verify accuracy of data collected by sites. Participants will receive a telephone call at each time point by research staff not involved in their care to ask their preference for follow-up. Unless she declines further participation, each participant will receive a reminder telephone call, and will be offered completion of the questionnaire by telephone if they feel they cannot complete it by mail or online.

#### Data management

Data collected will be entered into a registered electronic database by research staff blinded to treatment allocation who are not involved in the clinical care of participants. Hardcopies of participants’ data will be stored in a locked office. The electronic database will include the study identification number, but no directly identifying data such as medical record number, date of birth or personal address. The de-identified database will be backed up on a server at Royal Prince Alfred Hospital. Data linking identifying details to the study number will be kept at a separate location in a locked filing cabinet. At the end of the study, data will be kept in a locked filing cabinet, and de-identified electronic data will be kept on a portable medium, such as a USB drive, in a separate secure location at Royal Prince Alfred Hospital. All electronic data will be checked for accuracy by a second member of the research team, and any apparent data entry errors will be discussed by the primary investigators and investigated and/or corrected as required.

#### Analysis

Analysis will be on an intention-to-treat basis (according to treatment allocation), including withdrawals and losses to follow-up. Losses to follow-up for the primary outcome are not expected because randomisation will occur at full dilatation and the primary outcome is the mode of delivery. The results will be reported according to the Consolidated Standards for Reporting Trials (CONSORT) guidelines.

Demographics and other potential confounders will be compared by treatment allocation in a univariate analysis. Categorical outcome measures will be compared by proportions (chi-squared test), means for normally distributed data (t-test) or rank order for non-normally distributed data (Mann-Whitney U test).

A logistic regression analysis of treatment allocation and other variables on the primary outcome measure, operative delivery, will be performed. The following variables will be considered for the logistic regression model: maternal body mass index, maternal age, maternal height, maternal ethnicity, gestation, induction of labour, gestational diabetes, neonatal gender and RCOG CTG classification in the second stage of labour. Parity, study site and the presence of epidural for intrapartum analgesia at the time of randomisation will not be included because randomisation is stratified for these variables. Only variables where *P* <0.25 in the univariate regression will be included in the multivariate model. Continuous variables that do not show a linear association with the logit function will be divided into quartiles and treated as categorical. Interaction terms will be considered for treatment allocation versus each of the other variables and, where clinically appropriate, between non-treatment variables. *P* <0.01 will be considered evidence of interaction. Terms will be excluded from the model in a stepwise backward manner until all remaining terms are both statistically significant (*P* <0.05) and clinically significant (that is, removal of the term results in a clinically significant change in the estimate of the odds ratio of treatment allocation for the primary outcome). The analysis will be performed using SAS 9.2 (or a more recent version of SAS, Statistical Analysis Software. Cary, USA).

#### Additional analyses

Subgroup analyses will be performed according to the technique of manual rotation employed (manual/whole hand versus digital/fingers) and according to operator ability (data will be divided into two approximately equal groups according to the success rate of the operator who performed the manual rotation).

#### Data and Safety Monitoring Committee

Draft terms of reference for a Data and Safety Monitoring Committee provide for potential cessation of the trial if significant safety concerns are raised. The Data and Safety Monitoring Committee will consist of three people who are not involved in the study and do not have a working relationship with the primary investigators. Adverse events will be reported to the committee. Any serious complications will be referred to the Data and Safety Monitoring Committee. There will be no external auditing of the trial. This study has been approved by the Ethics Review Committee (RPAH Zone) of the Sydney Local Health District, Sydney, Australia (protocol number: X110410).

## Discussion

This trial addresses an important clinical question concerning a commonly used procedure that has the potential to reduce operative delivery and its associated complications. Due to the nature of the intervention, a number of issues are worthy of discussion.

First, empirical evidence suggests that blinding reduces bias in randomised controlled trials. However, blinding may be difficult in the case of procedural interventions. In this trial, we intend to assess the efficacy of blinding by asking the woman’s clinician to guess the treatment allocation after manual rotation or sham rotation has occurred. The purpose of this is to allow the reader to assess the risk of bias associated with knowledge of treatment allocation.

Second, the efficacy of procedural interventions may depend on the experience and training of individual operators. The success of manual rotation by individual operators will be assessed by recording the ultrasound-determined fetal position after the manual rotation or sham procedure has been performed. We will report on any major differences between the success rates of individual practitioners.

Third, due to the ethics of consent in labour, consent will be obtained when it is unknown if the fetus will be in the OT position in the second stage of labour, which is an eligibility criterion. Thus, it is likely that only a minority of consented participants will be randomised, which will result in a large workload per randomisation.

Finally, women who progress rapidly in labour may give birth before they can be randomised and women with regional analgesia will have more opportunity to be consented. This could result in the study population having a higher background risk of the primary outcome than non-consented women who meet our eligibility criteria, which could impact the generalizability of our findings.

### Protocol amendments and confidentiality

If modification to the study protocol is considered necessary then permission will be sought from the ethics committee and the changes will be described in the final report. All the information collected from the study will be treated confidentially, and only the researchers will have access to it. Hard copies of data collection forms will be stored in a locked office. The electronic database will be de-identified and stored at a different location to codes linking identifying data to study identification numbers. The electronic database will be on Microsoft Access, password-protected, and only accessible by research staff.

### Roles and responsibilities

#### Trial Management Committee

The committee consists of Hala Phipps, Jon Hyett and Bradley de Vries, who are responsible for the following:

1. Study planning,
2. Organisation of Steering Committee meetings,
3. Randomisation,
4. Reporting of any serious adverse events to the Data and Safety Monitoring Committee,
5. Budget administration and organising contracts with individual centres,
6. Providing advice for site investigators,
7. Auditing and visiting sites,
8. Data verification, and
9. Following up of study participants.

#### Site investigators and data manager

In each participating centre a lead investigator (obstetrician) will be responsible for identification, recruitment data collection and completion of relevant trial forms, along with adherence with study protocol. Each lead investigator will be a member of the Steering Committee. The data manger will be responsible for the maintenance of the trial IT system, and data entry and verification.

#### Steering Committee

The Steering Committee will be chaired by Brad de Vries and all lead investigators will be members of the Steering Committee, who are responsible for the following:

Recruitment of pregnant women on the study and liaising with principal investigators HP, JH and BD,

Reviewing progress of study and facilitating the smooth running of the trial,

Reporting the results of the trial.

## Trial status

The trial began recruitment on 2 May 2013 and has recruited 128 participants so far. Recruitment is expected to be completed by May 2019.

## Abbreviations

CI: Confidence Interval
CONSORT: Consolidated Standards for Reporting Trials
CTG: Cardiotocograph
Fig: Figure
ICU: Intensive Care Unit
NSW: New South Wales
NICU: neonatal intensive care unit
OA: occiput anterior
OP: occiput posterior
OR: Odds Ratio
OT: occiput transverse
RCOG: Royal College of Obstetrics and Gynaecology
RPAH: Royal Prince Alfred Hospital
SA: South Australia
SAS: Statistical Analysis Software
SF – 12: Short From – 12
TURN-OUT: occiput Transverse Using Rotation to aid Normal birth OUTcomes following manual rotation
USB: Universal Serial Bus
